# Emergency Surgery for Gastrointestinal Complications in Patients With Granulomatosis With Polyangiitis and Microscopic Polyangiitis

**DOI:** 10.7759/cureus.84496

**Published:** 2025-05-20

**Authors:** Masatoshi Inoue, Ryosuke Ichihara, Miya Hiramatsu, Junichiro Yamamoto

**Affiliations:** 1 Nephrology, Asahi University Hospital, Gifu, JPN; 2 Pathology, Tsushima City Hospital, Aichi, JPN

**Keywords:** antineutrophil cytoplasmic antibody-associated vasculitis, emergency surgery, gastrointestinal bleeding and perforation, granulomatosis with polyangiitis, microscopic polyangiitis

## Abstract

Gastrointestinal (GI) involvement in granulomatosis with polyangiitis (GPA) and microscopic polyangiitis (MPA) is rare but potentially life-threatening. We report two cases of GPA/MPA complicated by GI bleeding and perforation, both requiring emergency surgical intervention. The first case involved a man in his 30s with GPA who developed massive small bowel bleeding following treatment with high-dose corticosteroids and rituximab. The second case involved an elderly man with MPA, complicated by *Clostridioides difficile* enteritis and subsequent colonic perforation. These cases suggest that not only primary vasculitic vascular injury but also secondary factors, such as immunosuppressive therapy and opportunistic infections like *Clostridioides difficile, *may synergistically contribute to the development of severe GI complications. Surgical strategies must be carefully tailored based on disease activity, overall clinical condition, and corticosteroid exposure, with an emphasis on balancing the risks of rebleeding, infection, and healing failure in the context of systemic vasculitis. Emerging therapies such as avacopan may help mitigate the risk of GI complications. Early recognition and multidisciplinary management are essential to improving outcomes in patients with GI involvement in GPA/MPA.

## Introduction

Granulomatosis with polyangiitis (GPA) and microscopic polyangiitis (MPA) are systemic small-vessel vasculitides, often associated with positive antineutrophil cytoplasmic antibodies (ANCA) targeting either proteinase 3 (PR3) or myeloperoxidase (MPO). Both conditions can involve the kidneys, upper and lower respiratory tracts, eyes, peripheral nerves, skin, and central nervous system. However, central airway involvement is more characteristic of GPA, while interstitial pneumonia and bronchiectasis are more commonly observed in MPA [1]. Gastrointestinal (GI) involvement in ANCA-associated vasculitis is relatively uncommon, but when present, it can lead to serious complications such as bleeding or perforation. In an analysis of 378 patients with MPA, independent risk factors for mortality included age ≥65 years, serum creatinine ≥130 μmol/L, severe GI involvement, and polyangiitis, underscoring the clinical significance of GI lesions [2]. Nevertheless, whether these findings are due to vasculitis or therapy remains debated. Here, we report two cases of GPA and MPA complicated by GI lesions that necessitated emergency surgery - one presenting with gastrointestinal bleeding and the other with intestinal perforation. We highlight the clinical challenges in management and discuss implications for future treatment strategies.

## Case presentation

Case 1

A man in his 30s presented to our hospital with complaints of nausea, cough, and sore throat. He had a history of otitis media eight years earlier, at which time he was diagnosed with GPA. He was initially treated with prednisolone and cyclophosphamide (CYC), which was later switched to mizoribine. On admission, physical examination revealed edema of the entire upper extremities and fingers, with no edema in the lower extremities. His vital signs were as follows: body temperature 36.8°C, blood pressure 147/109 mmHg, heart rate 87 beats per minute, respiratory rate 15 breaths per minute, and oxygen saturation 99% on room air. A routine examination performed three weeks earlier had shown normal results, but at this presentation, his laboratory findings were markedly abnormal. Serum creatinine was elevated at 12.8 mg/dL (reference range: 0.46-0.79 mg/dL), C-reactive protein (CRP) was 22.9 mg/dL (reference range: <0.03 mg/dL), and PR3-ANCA was 64.6 IU/mL (reference range: <3.0 IU/mL) (Table 1). Whole-body computed tomography (CT) revealed bilateral renal enlargement without any pulmonary lesions. The Birmingham Vasculitis Activity Score (BVAS) version 3, 2008, was 13.

**Table 1:** Laboratory investigations of case 1. PR3-ANCA: proteinase 3-antineutrophil cytoplasmic antibodies

Variable	Reference range	On admission
Hemoglobin (g/dL)	11.6-14.8	11.5
Hematocrit (%)	35.1-44.4	33.3
White blood cell count (per μL)	3,000-8,600	22,100
Neutrophil count (per μL)	1,800-7,500	18,785
Lymphocyte count (per μL)	1,000-4,800	884
Eosinophil count (per μL)	100-300	0
Platelet count (per μL)	158,000-348,000	342,000
Creatinine (mg/dL)	0.46-0.79	12.8
Urea nitrogen (mg/dL)	8-22	89.3
Uinary acid (mg/dL)	3.7-7.8	20.5
Albmin (g/dL)	4-5	2.6
C-reactive protein (mg/dL)	0-0.3	22.2
Sodium (mmol/L)	138-146	134
Potassium (mmol/L)	3.6-4.9	5.1
Chloride (mmol/L)	99-109	99.7
Calcium (mg/dL)	8.6-10.2	9
Phosphorus (inorganic) (mg/dL)	2.5-4.5	5.2
Erythrocyte sedimentation rate (mm/h)	<10	105
PR3-ANCA (IU/mL)	<3	64.6

**Figure 1:**
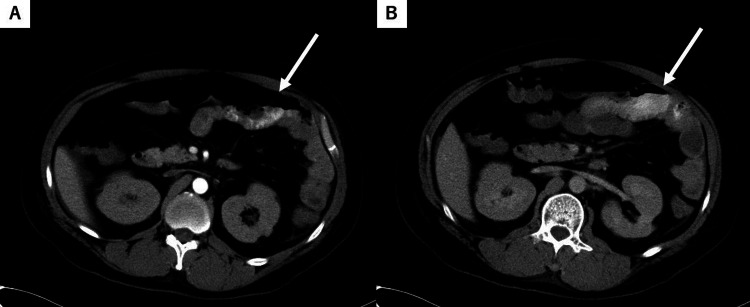
Contrast-enhanced computed tomography on day 23 for case 1. (A) Arterial phase image demonstrates a high-attenuation area in the jejunum, suggestive of active extravasation. (B) Equilibrium phase image shows persistent extravasation with increased distribution of contrast medium, consistent with ongoing small bowel hemorrhage.

**Figure 2:**
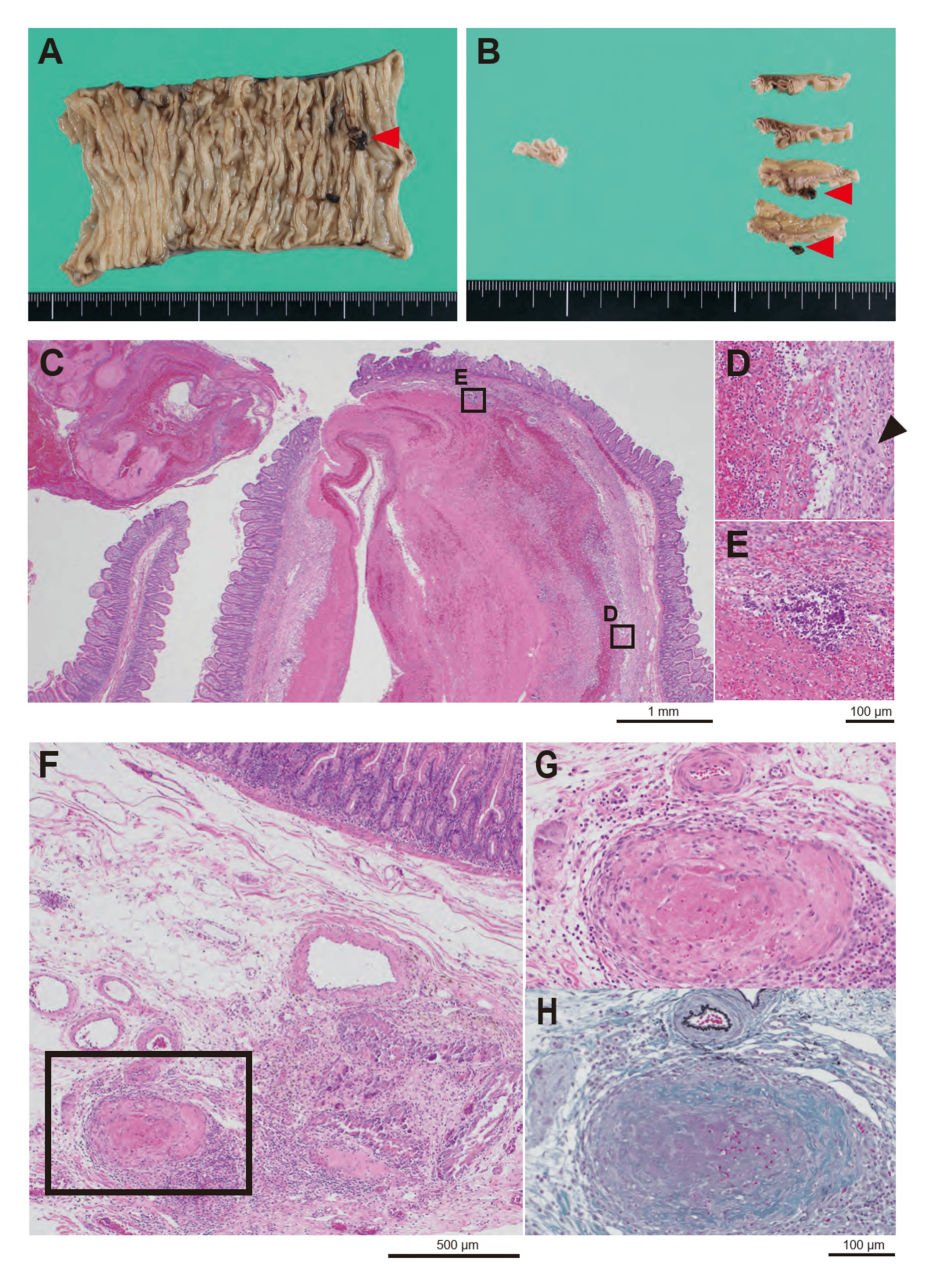
Pathological findings of the resected small intestine in case 1. The macroscopic appearance of the resected small intestine (A) and its cut surface (B) reveals a 5 mm mass lesion with black discoloration (red arrowheads). Histological findings of the mass lesion show extensive fibrinoid necrosis in the submucosa, some of which has destroyed the mucosa and is exposed on the mucosal surface, consistent with a source of hemorrhage. (C). At the margins of the necrotic lesions, an inflammatory cell infiltrate consisting mainly of neutrophils was observed (D and E), and granulomatous lesions consisting of histiocytic aggregates containing a small number of multinucleated giant cells (black arrowheads) (D) and microcalcified foci (E) are also seen at the necrotic margins. Necrotizing vasculitis is seen in the submucosa of the small intestine around the necrotic area (F, G: the rectangular area in F is G). Elastica Masson staining shows destruction of elastic fibers in the vessel wall (H).

The patient was hospitalized, and a vascular access catheter was placed to initiate hemodialysis. High-dose methylprednisolone (1,000 mg/day) was administered for three days, followed by oral prednisolone at 45 mg/day. On day 12, double filtration plasmapheresis was started and performed twice a week for a total of four sessions. Rituximab (RTX, 375 mg/m²) was initiated on day 20 and administered weekly for four doses. On day 23, the patient developed sudden abdominal pain accompanied by massive gastrointestinal bleeding. Emergency upper endoscopy and colonoscopy were performed but failed to identify the bleeding source. Contrast-enhanced CT revealed small bowel hemorrhage, prompting emergency surgery (Figures 1A, 1B). A 5 mm mass lesion was located 20 cm proximal to the ileocecal valve and was partially resected, followed by temporary anastomosis (Figures 2A, 2B). Histologically, vasculitis with fibrinoid necrosis and granulomatous changes was observed, which, when considered in conjunction with the clinical presentation, is consistent with granulomatosis with polyangiitis. (Figures 2C-2H). Despite surgery, bleeding persisted, and the patient required daily transfusions of six units of packed red blood cells. On day 29, he was transferred to a tertiary care center where endoscopic clipping of the small intestine was successfully performed. Bleeding was controlled, and the patient remained stable on maintenance dialysis with no recurrence of GI complications.

Case 2

**Figure 3:**
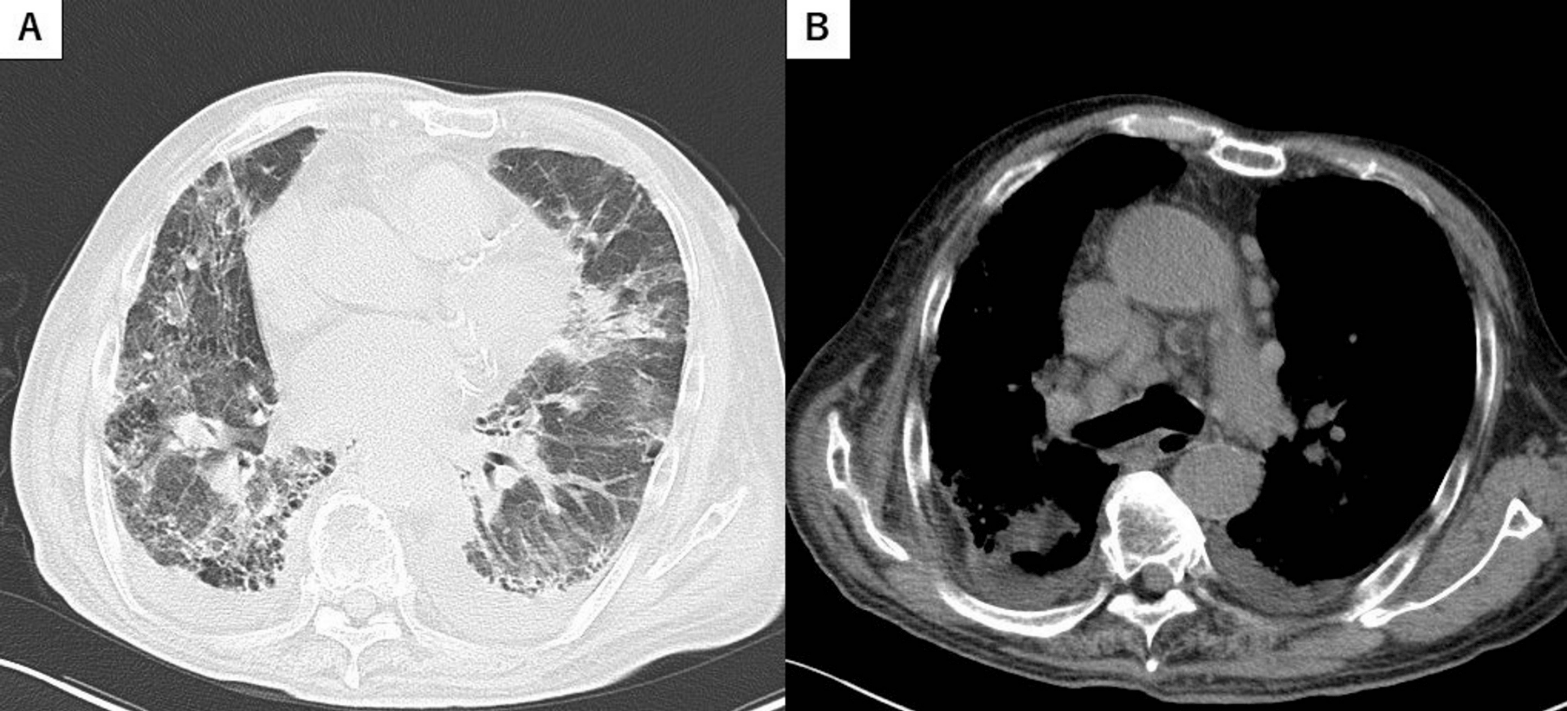
Computed tomography on admission of case 2. (A) Lung window image shows peripheral ground-glass reticular opacities, traction bronchiectasis, and cystic changes in both lungs, along with consolidation and pleural effusions in the upper and lower lobes. (B) Mediastinal window image reveals multiple enlarged mediastinal lymph nodes, including in the pretracheal and subaortic regions.

A man in his 80s initially visited his primary care physician with anorexia and weight loss. He was diagnosed with anemia and malnutrition and was managed conservatively for one month, but due to lack of improvement, he was referred to our hospital. On physical examination, he appeared pale and had conjunctival pallor, with no peripheral edema. Digital rectal examination revealed no signs of gastrointestinal bleeding. His body temperature was 37.0°C, blood pressure was 131 over 72 mmHg, heart rate was 68 beats per minute, and oxygen saturation was 97% on room air. Laboratory data showed serum creatinine 6.26 mg/dL, hemoglobin 9.0 g/dL (reference range: 11.6-14.8 g/dL), CRP 18.8 mg/dL, and MPO-ANCA >640 IU/mL (reference range: <3.5 IU/mL) (Table 2). Whole-body CT revealed bilateral ground-glass opacities and pleural effusions (Figure 3A, 3B). Echocardiography revealed an enlarged inferior vena cava (24 mm) and right ventricular dilation, suggestive of right-sided heart failure. The BVAS was 22.

**Table 2:** Laboratory investigations of case 2. MPO-ANCA: myeloperoxidase-antineutrophil cytoplasmic antibodies

Variable	Reference range	On admission
Hemoglobin (g/dL)	11.6-14.8	9.0
Hematocrit (%)	35.1-44.4	27.9
White blood cell count (per μL)	3,000-8,600	16,500
Neutrophil count (per μL)	1,800-7,500	14,570
Lymphocyte count (per μL)	1,000-4,800	677
Eosinophil count (per μL)	100-300	198
Platelet count (per μL)	158,000-348,000	200,000
Creatinine (mg/dL)	0.46-0.79	6.26
Urea nitrogen (mg/dL)	8-22	93.4
Uinary acid (mg/dL)	3.7-7.8	8.6
Albmin (g/dL)	4-5	3.1
C-reactive protein (mg/dL)	0-0.3	18.8
Sodium (mmol/L)	138-146	130.8
Potassium (mmol/L)	3.6-4.9	4.2
Chloride (mmol/L)	99-109	100.5
Calcium (mg/dL)	8.6-10.2	7.6
Phosphorus (inorganic) (mg/dL)	2.5-4.5	5.6
MPO-ANCA (IU/mL)	<3.5	>640

The initial diagnosis was severe bilateral pneumonia. Although the patient's SpO₂ was preserved, the risk of rapid deterioration was considered high, and meropenem was initiated on day one. After MPO-ANCA positivity was confirmed, high-dose methylprednisolone (500 mg/day for three days) was initiated on day six, followed by oral prednisolone at 45 mg/day. On day 26, the patient developed abdominal pain and tested positive for *Clostridioides difficile* (CD) toxin; oral vancomycin was administered. While symptoms temporarily improved, abdominal pain recurred on day 41. CT suggested perforation of the sigmoid colon, prompting emergency surgery. A fistula was closed, and a transverse colostomy was created. Despite surgical intervention, the patient’s respiratory and general condition worsened, and he died on day 53.

## Discussion

A review of 216 patients with GPA or MPA reported that 14 patients (6.5%) experienced gastrointestinal symptoms, with abdominal pain and GI bleeding being the most common manifestations. Among them, five patients required hemicolectomy or small bowel resection due to gastrointestinal perforation. The decision between performing a primary anastomosis and creating a temporary stoma is an important surgical consideration and is typically guided by steroid dosage and the patient's overall condition [2]. In the present report, both patients developed abdominal pain and required surgical intervention due to massive gastrointestinal bleeding and perforation. In case 1, a primary anastomosis was performed, whereas in case 2, a temporary stoma was created. The surgical approach in each case was selected based on the patient’s respiratory status, general condition, and BVAS. In case 1, rebleeding occurred near the anastomotic site and was managed endoscopically. Given the high-dose steroid therapy and the extensive necrosis observed on histopathology (discussed later), it is arguable that a temporary stoma may have been a more appropriate initial choice in this case. A study examining systemic small- and medium-vessel vasculitides, including GPA and MPA, reported a 10-month survival rate of 71% (95% confidence interval {CI}: 52-90%) and a five-year survival rate of 56% (95% CI: 35-77%) in patients who underwent surgical intervention. In contrast, patients who did not require surgery had significantly better outcomes, with a 10-month survival rate of 94% (95% CI: 87-101%) and a five-year survival rate of 82% (95% CI: 70-94%) [3]. These findings underscore the need for vigilant postoperative management, even if the patient survives the perioperative period without major complications. There have been reports of gastrointestinal bleeding or perforation occurring at different sites even after initial surgery, necessitating reoperation or additional endoscopic intervention [4]. In our case as well, the patient required massive red blood cell transfusions and further endoscopic treatment postoperatively. These findings highlight the systemic nature of vasculitis and the challenges involved in managing gastrointestinal complications associated with it.

In this report, the two cases of GPA and MPA were complicated by gastrointestinal bleeding and perforation, respectively. In case 1, severe GI bleeding occurred 23 days after intensification of immunosuppressive therapy, and in case 2, intestinal perforation occurred 35 days after initiation of treatment. The pathophysiology of vasculitis often involves vascular inflammation and ischemic changes, which may contribute to gastrointestinal complications. This suggests that immunosuppressive therapy itself could play a role in the development of such events. A disease-independent meta-analysis including 33,253 individuals reported a 43% increased risk of gastrointestinal bleeding or perforation associated with corticosteroid use (odds ratio: 1.43, 95% CI: 1.22-1.66) [5]. In our report, both patients received corticosteroids, which may have contributed to the development of GI bleeding and perforation. However, there are also reports of gastrointestinal hemorrhage as the initial presentation prior to the initiation of immunosuppressive therapy [6]. Therefore, GI complications may occur independently of treatment, suggesting a direct pathogenic role of vasculitis itself.

Of four cases of intestinal resection due to gastrointestinal perforation associated with GPA/MPA, vasculitis was identified in only two cases and granuloma in only one case, and pathology may not suggest a diagnosis or impact of vasculitis [2]. In case 1, necrotizing vasculitis was pathologically identified. GPA shows irregularly contoured lung necrosis, which is described as geographic necrosis [7]. However, the fibrinoid necrosis seen in the source of hemorrhage in case 1 was relatively well demarcated, so it was more likely to be a dilated and ruptured vessel caused by submucosal vasculitis rather than geographic necrosis. It is difficult to distinguish from histological findings alone whether the granulomatous changes seen at the necrotic margins are granulomas due to vasculitis of GPA or a reaction to vascular rupture.

Case 2 was complicated by CD enteritis. Toxins A and B produced by CD are known to damage the intestinal cytoskeleton, and when this occurs in areas of structural weakness, such as diverticula, it may lead to perforation [8]. In this case, resection of the intestine was not performed, and thus no pathological specimens were available. As a result, the exact mechanism of perforation remains unclear; however, a combined effect of vasculitis, CD enteritis, and glucocorticoid therapy may have contributed. At initial presentation, pulmonary lesions associated with MPA can be difficult to distinguish from those caused by infection. In such cases, broad-spectrum antibiotics are often administered empirically. However, the use of these agents may predispose patients to CD enteritis. In the context of vasculitis, CD enteritis can increase the risk of gastrointestinal bleeding or perforation. When infection has been ruled out, antimicrobial therapy should be promptly discontinued. Early diagnosis of vasculitis based on clinical presentation and laboratory findings is essential to avoid unnecessary antibiotic exposure and its associated complications.

Based on the results of a large randomized controlled trial, RTX is recommended over CYC in cases of severe relapse due to its superior efficacy and non-inferior safety profile [9]. In the present case, RTX was selected for treatment. Although the association between RTX and gastrointestinal bleeding remains unclear due to the lack of prior reports, no evidence currently suggests a significantly increased risk of GI bleeding with RTX use. Recently, avacopan, an oral complement C5a receptor inhibitor, has been approved and is increasingly used worldwide. When combined with CYC or RTX, avacopan has demonstrated efficacy in both induction and maintenance therapy for severe GPA/MPA, with the added benefit of potentially reducing or eliminating the need for glucocorticoids. Although the optimal patient population for avacopan use remains under investigation, its mechanism of action suggests that it may be particularly beneficial for patients with severe renal involvement or alveolar hemorrhage, as well as for elderly patients at high risk for steroid-related complications [10]. Case 2 involved an elderly patient with both severe interstitial pneumonia and impaired renal function, characteristics that may have made him a suitable candidate for avacopan therapy. With accumulating clinical experience, the appropriate use of avacopan is expected to contribute to improved outcomes and potentially save lives in high-risk vasculitis patients.

## Conclusions

In this report, we presented two cases of gastrointestinal bleeding and perforation in patients with GPA and MPA, both of which required surgical intervention. Although gastrointestinal involvement is relatively uncommon in ANCA-associated vasculitis, it can lead to life-threatening complications, underscoring the importance of early recognition and appropriate management. Further studies are needed to elucidate the underlying mechanisms and risk factors contributing to gastrointestinal complications in GPA/MPA. The use of avacopan, which allows for reduced or even eliminated glucocorticoid exposure, may reduce the risk of severe GI complications requiring surgery in the future.
